# Grafting and biodynamic nanosilica-induced physiological and transcriptomic modulation of chilli (*Capsicum annuum* L.) under drought stress

**DOI:** 10.3389/fpls.2026.1916999

**Published:** 2026-08-25

**Authors:** Nagarajan Bharathy, Theivasigamani Parthasarathi

**Affiliations:** 1 School of Biosciences and Technology, Vellore Institute of Technology, Vellore, Tamil Nadu, India; 2 VIT School of Agricultural Innovations and Advanced Learning, Vellore Institute of Technology, Vellore, Tamil Nadu, India

**Keywords:** *Capsicum*, drought, grafting, nano-silica, transcriptomics

## Abstract

Chilli (*Capsicum annuum* L.) is an economically important vegetable crop cultivated worldwide. Increasing drought stress associated with climate change has severely reduced chilli productivity. Although grafting and silicon-based nanomaterials have each been investigated independently as drought mitigation strategies in *Solanaceae* crops, this study represents, to our knowledge, the first investigation of their combined physiological, yield, and genome-wide transcriptomic effects in chilli under experimentally validated drought stress. Biodynamic nanosilica (BNS) is an α-quartz nanoparticle preparation (20–200 nm) derived from the biodynamic agricultural preparation BD501 through a vortex-triturating process, and distinct from chemically synthesised nanosilica in preparation method and surface bioavailability, applied as a foliar spray at 50 mg L^-1^. Five treatments were established: well-watered (WW), drought (D), grafting + BNS + drought (G+B+D), grafting + drought (G+D), and BNS + drought (B+D), each with three independent biological replicates. Under moderate-to-severe drought conditions (DSI 62–64%; VWC ~12% v/v at 14 days), the combined G+B+D treatment significantly improved plant height (3.05-fold over D), leaf relative water content (83% vs 49% in D), net photosynthetic rate (2.0-fold over D), water-use efficiency (+40%), and antioxidant enzyme activities (SOD: 3.1-fold; CAT: 2.8-fold over D), while reducing lipid peroxidation by 76%. Root architecture was also substantially enhanced, with a 4.1-fold increase in root length and a 3.1-fold increase in root surface area relative to D. Fruit yield increased by 79% relative to drought-stressed non-grafted plants. Transcriptomic analysis using Illumina NovaSeq 6000 identified 1,051 DEGs (431 upregulated, 620 downregulated; FDR < 0.05, |log_2_FC| > 1). Integrated transcriptomic–phenotypic concordance analysis revealed enrichment of MAPK signalling, ABA-mediated regulation (including ABA binding and (+)-ABA 8’-hydroxylase activity), and phenylpropanoid biosynthesis as the enriched pathways. Protein-protein interaction network analysis further revealed coordinated regulation of redox homeostasis, drought-responsive hormone signalling, and water transport gene modules in the combined treatment. These findings demonstrate that integrating grafting with biodynamic nanosilica is a promising strategy to enhance drought resilience and productivity in chilli, offering a sustainable approach for vegetable production under drought.

## 1 Introduction

Chilli (*Capsicum annuum* L.) plays a significant role as a fruit and vegetable worldwide. India has become the world’s largest producer, consumer, and exporter of chilli (Biswas et al., 2025). Chilli has potential economic importance worldwide because of its extensive use in culinary, medicinal and cosmetic applications. Assessing their economic importance, the use of chilli has significantly increased over the past few years, but it is susceptible to drought stress (Bhutia et al., 2018; Khaitov et al., 2019; Mahmood et al., 2021).

As a result of climate change, the global average temperature has increased significantly in recent years. Due to this, half of the world’s population is expected to face water scarcity by the end of 2050 (Mohsin et al., 2025; Shemer et al., 2023). Agriculture accounts for approximately 70% of global freshwater withdrawal (Kay et al., 2022; Mishra, 2023). Drought is a primary abiotic stress that adversely affects plant growth and development (Taheri et al., 2026). As of early 2024, 26% of India’s land area was affected by drought, more than double that of the previous year (Charak et al., 2024). Consequently, chilli production in India has been adversely affected over the past year by 10-13.2% yield losses (Sakthivel and Reddy, 2024). Drought decreases plant growth, chlorophyll pigments and impairs physiological activity (Qiao et al., 2024; Zahra et al., 2023; Siddique et al., 2000). Also, it causes oxidative damage and reduces fruit set, weight, yield and quality (Dietz et al., 2021; Liu et al., 2023; Medyouni et al., 2021; Rao et al., 2025) and Depending on the intensity and duration of stress, it can cause damage or death to plants. Therefore, sustainable agricultural production strategies are becoming increasingly challenging.

Numerous techniques have been applied to increase plant resistance to drought stress. One of these techniques is grafting, which is a rapid, ancient, sustainable, and highly effective technique for improving drought tolerance in plants, such as chilli (Dogan et al., 2025a; Padilla et al., 2024; Singathiya et al., 2025). It is a speedy alternative to moderately slow breeding techniques. Grafting involves uniting two different plants or plant parts in such a way that their vascular systems are joined and grow together as a single plant exhibiting the characteristics of both the parent plants (Feng et al., 2024). It is widely used in commercial vegetable and fruit production, particularly in the *Solanaceae* and *Cucurbitaceae* families. It is widely used to mitigate the effects of abiotic stresses and increase yield and fruit quality (Bahadur et al., 2024; Dogan et al., 2025b). It has a positive effect by increasing root biomass and water absorption capacity; conserving water via stomatal regulation; activating osmotic and antioxidant defences; promoting beneficial molecular signalling between rootstock and scion; and sustaining growth and photosynthesis under water stress (Lal et al., 2025; Liu et al., 2023).

Similarly, nanosilica has a potential role in enhancing plant development, especially crop productivity, during abiotic stress. This greatly increases plant resistance to drought stress (Daler et al., 2024; Sharma et al., 2024; Panigrahi and Rout, 2025). Many types of nanosilica have been used in plants. BNS is prepared by the biodynamic vortex-triturating process applied to quartz-based BD501 preparation, yielding α-quartz nanoparticles with a crystalline microflake structure (20–200 nm). BD501 most commonly refers to Horn Silica, a vital biodynamic preparation used in organic farming. It is typically applied to foliage during blossoming, fruit set, and pre-harvest. This nano preparation method of BD501 differs fundamentally from conventional chemical synthesis routes (e.g., sol-gel or Stöber processes), which rely on synthetic reducing agents or controlled hydrolysis, and may confer distinct surface properties and bioavailability characteristics relevant to foliar uptake and plant interaction (Bharathy and Parthasarathi, 2025). However, its role in plant drought stress responses remains largely unexplored.

Although, grafting and nanosilica have independently been shown to enhance drought tolerance in plants, limited information is available regarding their combined effects and the associated physiological and molecular responses, particularly in chilli under drought stress conditions. Given these facts, the present study aimed to investigate the performance of grafting susceptible *Capsicum* scions (Samba variety) onto drought-tolerant rootstocks (Kanthari variety), combined with the application of an optimal concentration of BNS. Therefore, the objectives of this study were to evaluate the influence of grafting and BNS application on the morphological and physicochemical parameters of chilli plants and to determine the effect of grafting and BNS supplementation on the yield and fruit quality of chilli. Furthermore, we analysed drought-induced molecular interaction influenced by grafting and nanosilica (BNS) applications in chilli crops using transcriptomic analysis.

## 2 Materials and methods

### 2.1 Experimental setup

This study was conducted in an experimental field greenhouse of the VAIAL, Vellore Institute of Technology (12°58’ 9.12’ N, 79°09’ 21.24’ E). The “Samba” variety was used as a scion, and the “Kanthari” variety was used as rootstock. The seeds of the rootstock and scion were sown in a tray filled with coir, peat and vermicompost at a 1:1 ratio. Three days later, the seeds of the scion were sown in a manner similar to that of the rootstock seeds. Three-week-old seedlings were subjected to splice grafting, where the shoot positions of the rootstock and scion were cut off at a 45° angle. The shoots of the scion and rootstock were placed in such a way that their vascular systems were aligned using a grafting clip. The grafted plants were kept undisturbed in the shade and covered with a polythene cover for ten days to develop a successful graft union. During the wound-healing process, the plants were maintained at 21–25 °C, with a relative humidity of approximately 70–85%. After ten days, the grafting clips were removed, and the grafted plants were grown under normal day/light conditions for hardening. The 20 DAG-old grafted plants were transferred to pots (4x4 inch, 2–3 kg soil-holding capacity) with a growing substrate consisting of red soil and vermicompost in a 2:1 ratio.

The experimental setup (**Supplementary Figure 1**) designed in a factorial completely randomised design consists of five treatments with three replicates per treatment, namely, non-grafted scion without Biodynamic Nano Silica (BNS) in well-watered (WW), non-grafted scion without BNS in drought (D), grafted with BNS under drought (G+B+D), grafted without BNS under drought (G+D), non-grafted scion with BNS under drought (B+D). Each replicate consisted of one plant grown in an individual pot. BNS was procured from the VIT School of Agricultural Innovations and Advanced Learning (VAIAL), Vellore Institute of Technology and the effective concentration was determined by preliminary screening. The effective BNS foliar spray concentration of 50 mg L^-1^ was determined through preliminary screening across four concentrations (0, 25, 50, and 100 mg L^-1^), with 50 mg L^-1^ yielding the highest plant biomass, pigments and physiological performance. BNS was applied to the G+B+D and B+D treatments by foliar spray three days prior to the drought induction. Drought stress was induced on the vegetative (30 DAG) and reproductive stages (70 DAG) of the plant by the withholding method for 14 days. Pots were initially saturated to field capacity (~35% v/v), determined gravimetrically using a red soil:vermicompost (2:1) mixture (Earl, 2003). Before drought imposition, all pots were uniformly irrigated to field capacity (~35% v/v soil moisture), and the initial soil water status was maintained under identical greenhouse and irrigation conditions across treatments. Drought stress was then initiated simultaneously by withholding irrigation from the designated treatments and soil water status was monitored throughout the experiment. To account for the possibility that treatment-dependent differences in plant size, leaf area, root development, and transpiration rate could cause unequal rates of soil water depletion across pots, daily pot weighing was used to track water loss dynamics throughout the drought period. All drought-assigned pots were brought to identical field capacity (~35% v/v VWC) on the day drought was imposed, and irrigation was withheld simultaneously across all treatments. DSI, VWC, and ψ were recorded at both 7 and 14 days of drought imposition and subjected to statistical comparison across treatments. The FTSW–NTR was additionally applied to characterize the transpiration response of each treatment relative to its own soil water depletion, thereby provided a plant-size-independent validation of drought intensity equivalence across treatments.

The drought stress index (DSI) was calculated using the method of Earl (2003): DSI (%) = [1 − (Wt − Wd)/(Wi − Wd)] × 100, where Wt is the pot weight at time ‘t’, Wi is the initial saturated weight, and Wd is the dry soil weight. Soil volumetric water content (VWC) was estimated from gravimetric measurements, and soil matric potential (ψ) was monitored using ceramic-cup tensiometers. Soil matric potential was monitored using a ceramic-cup tensiometer installed in a pot per treatment. Measurements were recorded at regular intervals during the drought period to track general soil drying trends.

### 2.2 Morphological and physiology parameters

After 14 days of drought stress, morphological parameters such as plant height, leaf count, and leaf area were measured. Plant height was measured using a measuring tape, and leaf area was calculated using the formula: 0.63 × length × breadth × number of leaves (Ray and Singh, 1989).

The non-destructive physiological parameters, namely photosynthetic rate (Pn), stomatal conductance (Gs), transpiration rate (E), photosystem II yield, and water use efficiency (WUE), were measured on the 7th (37 DAG) and 14th (45 DAG) days of drought treatment. Photosynthetic rate and stomatal conductance were measured using a portable photosynthesis system (LI-COR 6800). Green leaves were enclosed in a portable photosynthesis system under a light intensity of 800 µmol m^-2^ s^-1^ PPFD and 400 µmol mol^-1^ CO_2_ at 25 °C leaf temperature and relative humidity between 40 and 55%. A Mini-PAM 2000 with a leaf holder was used to measure non-photochemical quenching (NPQ) in the leaves. The intrinsic water use efficiency was calculated using the formula, WUE (µmol CO_2_ mmol^-1^ H_2_O) = Pn/gs.

Drought intensity was physiologically validated using the FTSW–NTR approach (Belko et al., 2012). Pot weights were recorded daily during the dry-down period to calculate the fraction of transpirable soil water (FTSW), where FTSW = (daily weight − final weight)/(initial weight − final weight). Initial weight was defined as the pot weight at field capacity at the start of drought imposition, and final weight was the pot weight at the end of the drying cycle. Daily transpiration was used to compute the transpiration ratio (TR) of stressed plants relative to the mean transpiration of the control plants. TR values were subsequently normalized to obtain the normalized transpiration rate (NTR). The FTSW–NTR relationship was used to characterize the physiological onset of drought stress and compare treatment-specific transpiration responses during progressive soil drying.

Relative water content was measured in physiologically active leaves using the modified method described by Nir Sade et al. (2015). The entire fresh leaves were weighed (fresh weight, FW) and placed in a plastic bag with distilled water such that the petiole was immersed in water. After eight hours, the turgid leaves were removed, and the surface water was removed and weighed (turgid weight, TW). The leaves were then dried in an oven at 60 °C for two days. The dried leaves were weighed (dry weight, DW), and finally the RWC is calculated using the formula: RWC = (FW-DW/TW-DW) × 100.

### 2.3 Sample collection

Leaf samples were collected after 14 days of stress (45DAG) during the vegetative stage. It was stored at -20 °C for further biochemical analysis (photosynthetic pigment, phenol, total soluble sugars, proline, enzyme activity, and stress markers). Leaf samples for transcriptomics were also collected and stored at -80 °C. Fully developed mature green chillies were harvested at 84 DAG, with four pickings at intervals of 10–15 days. The fruit samples from the four pickings were sampled together for further analysis.

### 2.4 Biochemical assessment

Biochemical and antioxidant enzyme assays were performed using leaf samples from independent biological replicates (n=3). Total phenolic compounds were determined according to the method described by Sadasivam and Manickam (2008). A 1 g fresh leaf sample was ground with 10 ml of 70% acetone to obtain the plant extract. A total of 0.5 ml of 10% Folin-Ciocalteu reagent was added to 1 ml of plant extract, followed by 2 ml of 7.5% sodium carbonate. The mixture was then incubated in the dark for 30 min. The absorbance was measured at 765 nm. The total phenol content was calculated and expressed as mg gallic acid equivalents (GAE) per gram of fresh weight (FW).

Total soluble sugar content was determined using the anthrone method (Sadasivam and Manickam, 2008). Fresh samples (0.5 g) were ground with 80% ethanol and centrifuged at 10,000 rpm for 10 min. The supernatant was collected, and 4 ml of 0.2% anthrone reagent was added to 1 ml of the extract. The solution was gently mixed and kept in a water bath for 10 min at 70 °C. The absorbance of the green solution was measured at a 620 nm wavelength.

The photosynthetic pigments chlorophyll a and chlorophyll b were determined using the acetone method (Narwal et al., 2007). Fresh leaves (0.5 g) were ground in 10 ml of acetone. The solution was centrifuged at 5000 rpm for 10 min. The clear filtrate was used to measure the absorbance at 663 nm and 645 nm using a UV-Vis spectrophotometer.

Chlorophyll *a* (mg/g) = ((12.7 x A_663_ - 2.69 x A_645_) x V)/1000 x W
Chlorophyll *b* (mg/g) = ((22.9 x A_645_ – 4.68 x A_663_) x V)/1000 x W

where A_663_ is absorbance at 663 nm, A_645_ is absorbance at 645 nm, V is volume of extract (ml), W is fresh weight of leaves, and 1000 is conversion factor.

Proline was determined by the UV spectroscopic method (Chen and Zhang, 2016). A 0.5 g leaf sample was ground with 3% sulfosalicylic acid and centrifuged at 10000 rpm for 10 min. The supernatant was collected to determine proline content using UV spectroscopy. To 1 ml of supernatant, 2 ml of 2.5% ninhydrin and 2 ml of glacial acetic acid were added and incubated in boiling water (100 °C) for 60 min. The solutions were cooled immediately, and 4 ml of toluene was added to separate the chromophore layer. The coloured toluene layer was used to measure proline at 520 nm. It was expressed in µmol/g of fresh weight and calculated using a proline standard curve.

Malondialdehyde (MDA) was determined by the TBA (Thiobarburturic Acid) method (Chen and Zhang, 2016), which measures lipid peroxidation by reacting MDA with TBA to form a pink adduct. A 0.5 g fresh leaf sample was homogenised in 0.1% (w/v) trichloroacetic acid (TCA). The homogenised sample was centrifuged at 10000 rpm for 10 min at 4 °C to collect the clear supernatant. To 1 ml of sample, 0.5% thiobarburturic acid and 0.01% butylated hydroxytoluene were added and incubated at 95 °C for 50 min. After incubation, the temperature was immediately decreased to 25 °C, and the samples were centrifuged at 10000 rpm for 10 min. The absorbance was measured at 532 and 600 nm, and MDA was calculated using the formula MDA (nmol/g of FW) = 155 × (A_532_ - A_600_) × V/W × 10³, where A is absorbance, V is total extract volume, and W is fresh weight.

The enzyme extract was prepared by homogenising the fresh leaf samples with a 50 mM phosphate buffer solution (PBS) and centrifuged at 12000 rpm for 15 min. The supernatant was collected and used to determine the enzyme activity. All procedures were performed in a cooled condition (4 °C).

The SOD activity of chilli plants under drought conditions was determined using the modified method of Chen and Zhang (2016). The reaction mixture (for one reaction) contained 1.5 ml of 50 mM PB, 0.3 ml of 13 mM methionine, 0.3 ml of 75 µM NBT, 0.05 ml of 0.1 mM EDTA, and 0.3 ml of 2 µM riboflavin. To 0.1 ml of plant extract, 3 ml of the reaction mixture was added. The tubes were kept in light at 25 °C for 30 min and stored in the dark to measure the absorbance at 560 nm. The tube without plant extract served as the control. The inhibition percentage was calculated using the absorbance value, followed by SOD activity calculation, and expressed as U/g FW.

The CAT reaction was observed by adding 2.5 ml of 50 mM buffer, 0.4 ml of 10 mM H_2_O_2_ and 0.1 ml of extract (Chen and Zhang, 2016). Immediately, a decrease in absorbance was noted for 1 min at 15 s intervals at the 240 nm wavelength. The CAT activity was calculated as CAT (µmol/min/g FW) = (ΔA_240_/min) × Vt/(ϵ×l×Vs), where ΔA_240_ is change in absorbance per min at 240 nm, Vt is reaction volume, ϵ is 39.4 M^-1^ cm^-1^ (extinction coefficient for H_2_O_2_), l is path length of the cuvette, and Vs is sample extract volume.

The POD reaction mixture consisted of 2.5 ml of PBS, 0.3 ml of 20 mM guaiacol, and 0.1% of 20 mM H_2_O_2_ added together. To determine POD activity (Chen and Zhang, 2016), the reaction mixture was added to a cuvette, and 0.1 ml of plant extract was added. The change in absorbance was measured at 470 nm for 2 min at 30 s intervals. POD activity was calculated using POD (µmol/min/g FW) = (ΔA_470_/min) × Vt/(ϵ × l × Vs), where ΔA_470_ is change in absorbance per min at 470 nm, Vt is reaction volume, ϵ is 26.6 x 10³ M^-1^ cm^-1^ (extinction coefficient), l is path length of the cuvette, and Vs is sample extract volume.

To quantify APX activity (Law et al., 1983), 2.5 ml of 50 mM PBS buffer, 0.1 ml of 0.1 mM ascorbic acid, 0.1 ml of plant extract, and 0.1 ml of 0.1 mM H_2_O_2_ were added to a cuvette. A decrease in absorbance was observed at 290 nm for 2 min. The APX activity was calculated as follows: APX (µmol/min/g FW) = (ΔA_290_/min) × (Vt/ϵ×l×Vs), where ΔA_290_ is change in absorbance per min at 290 nm, Vt is reaction volume, ϵ is 2.8 × 10^4^ M^-1^ cm^-1^ (extinction coefficient for ascorbate), l is path length of the cuvette, and Vs is sample extract volume.

Antioxidant enzyme activities were calculated and expressed on a fresh weight basis (U g-1 FW). Reporting per unit fresh mass provides a direct measurement of net operational ROS-scavenging capacity across functional leaf tissue, bypassing analytical distortion caused by stress-induced protein degradation, dynamic protein turnover, and phenolic assay interference under progressive drought.

A 0.5 g leaf sample was added to 2.5% NaOH and incubated for 1 h at 95 °C. The digested sample was cooled, and the pH was adjusted to 2.5 by adding HCl. The solution was prepared to a final volume of 50 ml using distilled water. To determine the silica concentration in the plants, 1 ml of 10% oxalic acid was added to 5 ml of the digested extract, followed by the addition of 5 ml of 5% ammonium molybdate, and mixed for 5 min. Then, 1 ml of ascorbic acid was added and mixed well. The cells were then incubated for 30 min in the dark, and the absorbance was measured at 810 nm (Boone, 2007).

### 2.5 Yield parameters

Fruit yield parameters, such as fruit count per plant, fruit weight per plant, fruit length, fruit diameter, fruit shape index, fruit volume, fruit fresh weight, and fruit dry weight, were measured. Root architecture was examined at the time of plant harvest (120DAG). Root architecture analysis was performed using the WIZRHIZO root scanner software pro with a specialised Epson scanner.

### 2.6 Transcriptomic analysis

Total RNA was isolated from leaf samples (n=2 biological replicates per treatment; G+B+D, D) collected at 45 DAG (14 days drought) using the TRIzol method and quantified with NanoDrop 2000. RNA integrity was assessed using Agilent Bioanalyzer (RIN >7.0). cDNA libraries were prepared using KAPA HyperPrep Kit from 1 μg total RNA, with fragment sizes verified (200–500 bp) on Bioanalyzer. Libraries underwent paired-end sequencing (2×150 bp) on Illumina NovaSeq 6000, yielding 51.8 million reads per sample (7.81 Gb for drought treatment; **Supplementary Table 1**).

Raw reads were quality-trimmed (Trimmomatic v0.39; Q<20, adapters removed) and mapped to Capsicum annuum reference genome (v2.0) using HISAT2 (95.2% overall mapping rate; G+B+D 92.55%, D 93.53% raw data). Read counts were generated with featureCounts (v2.0.1). Differential expression analysis used DESeq2 (v1.34.0) in R (v4.2.1) with FDR <0.05, |log2FC| >1 thresholds, comparing G+B+D vs D treatments. Genes with >10 normalized counts in ≥2 replicates were retained.

Gene Ontology (GO) and KEGG pathway enrichment used clusterProfiler (v4.4.4) (FDR <0.05). Protein-protein interaction (PPI) networks of DEGs were constructed in STRING database (v12.0; medium confidence score 0.4) and visualized in Cytoscape (v3.10.4). GO Molecular Function enrichment was performed via STRING (FDR <0.05).

### 2.7 Statistical analysis

All morphology, physiology, biochemical, yield data were subjected to two-way analysis of variance (ANOVA) and using JMP 19. The mean values were compared using Tukey’s test (p < 0.05). Furthermore, a correlation matrix was used to assess the correlation between physiology and yield parameters of treatments.

## 3 Results

### 3.1 Drought stress validation: soil water status and stress uniformity across treatments

Drought progression was quantified using DSI, VWC, and soil matric potential (**Table 1**). At 7 days of drought imposition (37 DAG), DSI ranged narrowly from 26.7% to 29.7% across all drought-assigned treatments, with no significant differences among them (p > 0.05, Tukey’s test). By 14 days (45 DAG), DSI had increased to 62.4–64.1%, corresponding to a VWC of approximately 12% v/v and soil matric potentials of −65 to −68 kPa across all drought treatments. No significant differences in DSI, VWC, or ψ were detected among drought treatments at either time point, confirmed that all plants were subjected to comparable levels of moderate-to-severe drought stress irrespective of treatment-associated differences in plant size or transpiration demand. A potential concern is that larger or more physiologically active plants (e.g., grafted treatments, which showed greater leaf area and transpiration) could have depleted soil water faster in absolute terms, thereby experiencing greater drought intensity than smaller non-grafted plants. However, the FTSW metric normalizes water status against each pot’s own field-capacity baseline, made inter-treatment comparisons independent of absolute plant size or transpiration rate. The convergence of DSI, VWC, and ψ across treatments at both 7 and 14 DOD therefore confirmed that equivalent fractions of available soil water had been depleted in all drought pots at the time of physiological sampling, and that observed treatment differences in plant responses reflect biological effects of grafting and BNS.

**Table 1:** Soil moisture dynamics indicating comparable drought stress levels across treatments at 7 and 14 days of drought imposition (37 and 45 DAG).

Treatments	7 DOD DSI (%)	14 DOD DSI (%)	14 DOD VWC (% v/v)	14 DOD ψ (kPa)
WW	2.7 ± 0.8c	2.3 ± 0.2b	28.5 ± 1.2	-15
D	28.1 ± 1.1a	62.8 ± 1.0a	12.5 ± 0.8	-65
G+B+D	29.4 ± 0.5a	64.1 ± 1.4a	12.0 ± 0.6	-68
G+D	26.7 ± 0.2b	63.1 ± 1.8a	12.3 ± 1.0	-66
B+D	29.7 ± 0.8a	62.4 ± 0.3a	12.6 ± 0.5	-65

WW, well-watered; D, drought; G+B+D, grafted + BNS + drought; G+D, grafted + drought; B+D, BNS + drought. Values represent mean ± SE (n=3). Different letters indicate significant differences at *p* < 0.05 (Tukey’s test). VWC derived from DSI using field capacity 35% v/v.

The FTSW–NTR analysis revealed treatment-dependent transpiration dynamics during progressive soil drying, provided a validation of drought intensity across treatments (**Supplementary Figure 2**). In the D treatment, NTR declined progressively from approximately 1.05 at high FTSW (~1.0) to approximately 0.30 at near-zero FTSW, followed a strong negative linear relationship (R^2^ = 0.923). This pattern reflects a gradual stomatal and hydraulic restriction of transpiration as soil water availability decreased, characteristic of drought-sensitive non-grafted plants. The B+D treatment showed a similar declining NTR trajectory (R^2^ = 0.926), with NTR decreasing from approximately 0.95 to 0.75 over the same FTSW range, indicated that BNS application alone modestly buffered but did not reverse the progressive reduction in transpiration under drought. In contrast, grafted treatments exhibited different FTSW–NTR relationships. In G+B+D, NTR increased with declining FTSW (R^2^ = 0.940), raised from approximately 0.90 at high FTSW to values exceeding 1.2 at low FTSW. Similarly, G+D showed an increasing NTR trajectory with declining FTSW (R^2^ = 0.943), with NTR ranging from approximately 0.90 to 1.2 as FTSW approached zero. The NTR values consistently exceeding 1.0 in both grafted treatments indicate that grafted plants sustained transpiration rates above the well-watered control mean as soil water depleted, reflected rootstock-conferred hydraulic advantages that maintained stomatal aperture and leaf hydration despite declining soil water availability. The strong regression fits across all four treatments (R^2^ = 0.923–0.943) confirmed that transpiration responded consistently to progressive soil drying in all treatment groups, validated the physiological integrity of the drought imposition protocol.

### 3.2 Shoot growth and root architectural responses under drought stress

**Figure 1:**
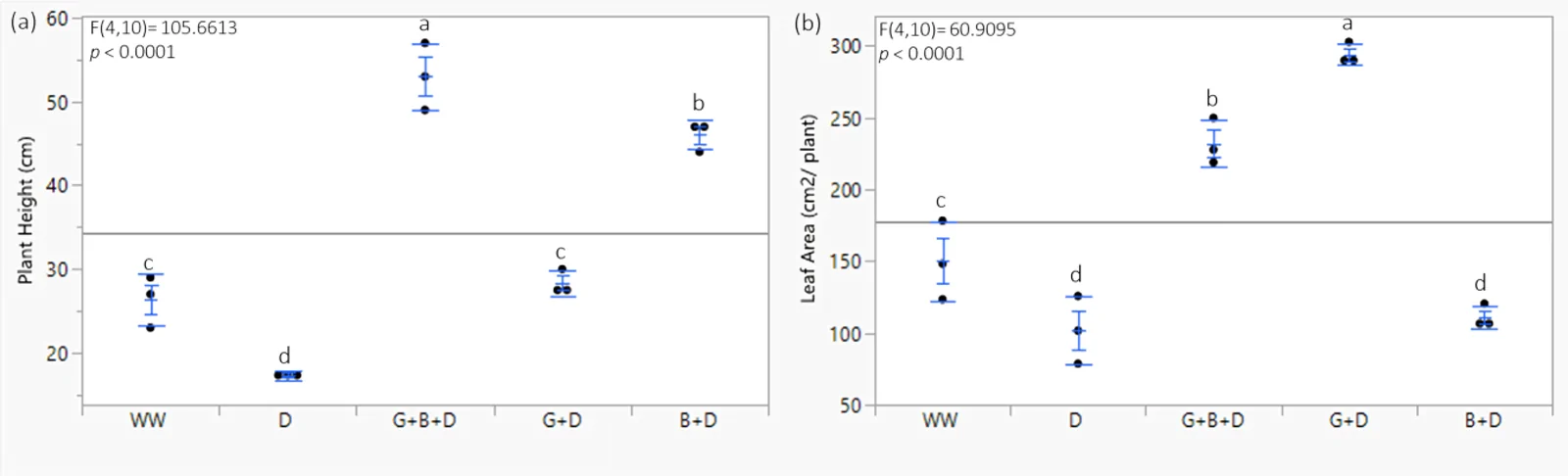
Effects of grafting and biodynamic nanosilica on the morphology of chilli plants under drought stress. Dot plots show individual replicates (n = 3) and mean ± SE for **(a)** plant height and **(b)** leaf area across treatments: well-watered (WW), drought (D), grafted + nanosilica + drought (G+B+D), grafted + drought (G+D), and nanosilica + drought (B+D). Different letters indicate significant differences at *p* < 0.05 (Tukey’s test). Observations were recorded on 45 DAG.

Plant height and leaf area were significantly different among the treatments (**Supplementary Figure 3**). Compared with WW, plant height in D was reduced by 34.1%, whereas G+B+D, G+D, and B+D increased plant height to 53.0 cm, 28.3 cm, and 46.0 cm, respectively, representing 3.05-, 1.63-, and 2.65-fold increases over D (**Figure 1a**). Similarly, leaf area was 2.27- and 2.88-fold higher in G+B+D and G+D than in D, respectively (**Figure 1b**).

Drought stress significantly altered root architecture in all treatments (**Supplementary Figure 5**). Drought stress decreased root length by 68% in the D treatment compared to the WW treatment, but increased in G+B+D to 1643.2 ± 95.8 cm, a 4.1-fold increase over D. Root surface area also increased from 67.8 ± 12.6 cm^2^ in D to 212.3 ± 6.9 cm^2^ in G+B+D (3.1-fold). Root complexity increased from 2,114.7 in D to 13,394.7 in G+B+D and G+D, while B+D showed the highest root tip number (5,162.7) (**Table 2**).

**Table 2:** Influence of grafting and BNS on root parameters of *Capsicum annuum* varieties grown under different treatments.

Treatments	Length(cm)	Surface area (cm^2^)	Length per volume (cm/m^3^)	Root volume (cm^3^)	Tips	Forks	Crossings
WW	1261.5 ± 212.7ab	151.3 ± 26.7ab	1261.5 ± 212.7b	1.4 ± 0.3abc	1960.7 ± 228.4b	8681.0 ± 1760.6a	1922.3 ± 394.6ab
D	395.7 ± 14.8c	67.8 ± 12.6b	395.7 ± 14.8c	1.0 ± 0.3bc	972.3 ± 368.9b	2114.7 ± 208.3b	298.3 ± 22.4c
G+B+D	1643.2 ± 95.8a	212.3 ± 6.9a	1643.2 ± 95.8a	2.2 ± 0.0a	1781.0 ± 177.4ab	12367.3 ± 682.8a	2790.0 ± 261.6a
G+D	1862.2 ± 198.9a	238.9 ± 32.3a	1862.2 ± 198.9ab	2.5 ± 0.4ab	3513.7 ± 1185.3b	13394.7 ± 1593.9a	2928.3 ± 499.4a
B+D	681.8 ± 43.2bc	79.7 ± 2.0b	681.8 ± 43.2c	0.8 ± 0.1bc	5162.7 ± 645.2a	3258.3 ± 340.8b	567.0 ± 97.7bc

WW, well-watered; D, drought; G+B+D, grafted + BNS + drought; G+D, grafted + drought; B+D, BNS + drought. Mean ± standard error followed by same letter are not significant differences at *p* < 0.05 (Tukey’s test). Observations recorded on 120 DAG.

### 3.3 Photosynthetic performance, gas exchange, and water relations under drought

**Figure 2:**
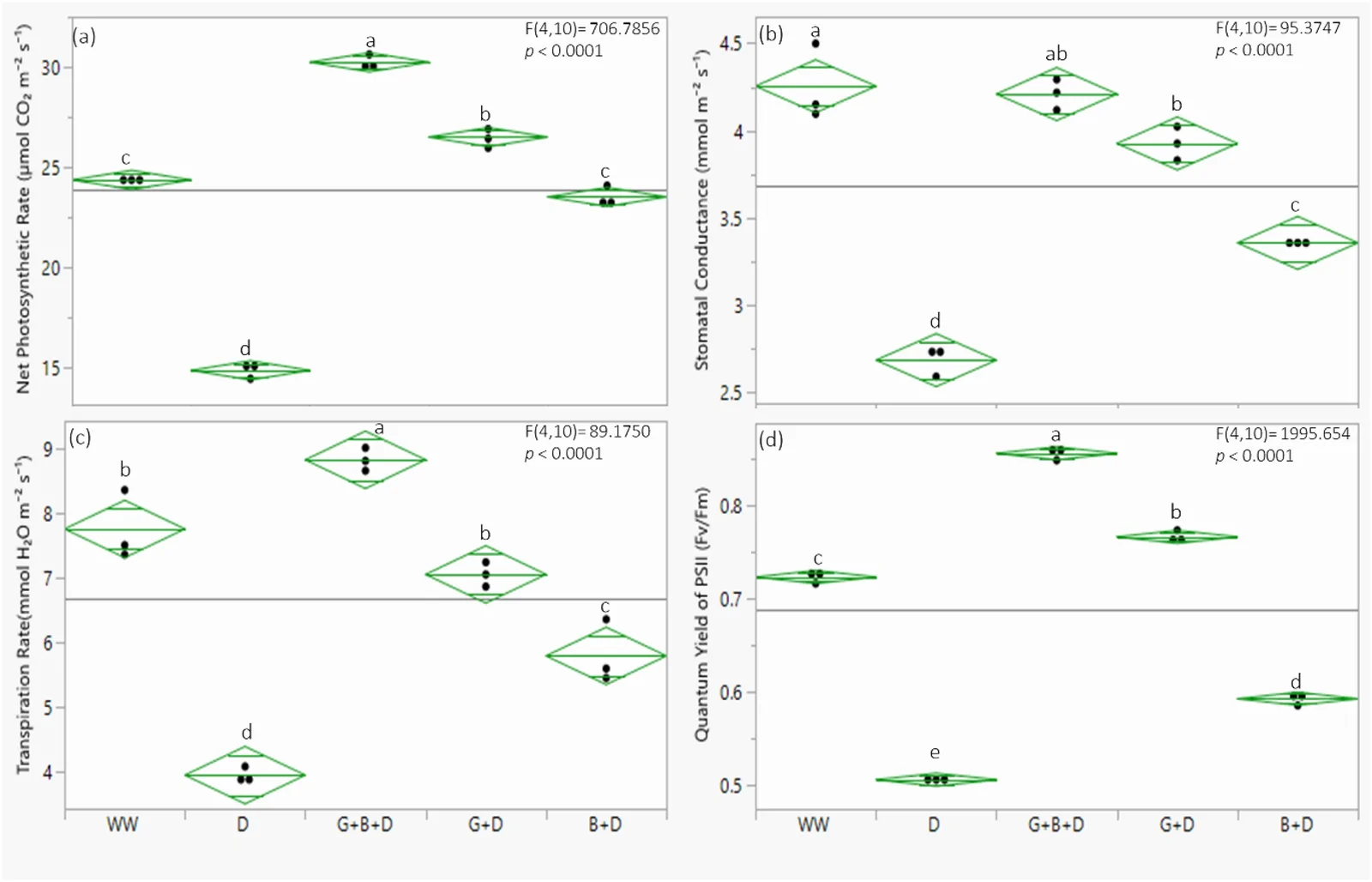
Violin plots showing the effects of grafting and biodynamic nanosilica on **(A)** net photosynthetic rate, **(B)** stomatal conductance, **(C)** transpiration rate, and **(D)** PSII efficiency (Fv/Fm) in chilli plants under drought stress. Treatments include well-watered (WW), drought (D), grafted + BNS + drought (G+B+D), grafted + drought (G+D), and BNS + drought (B+D). Black dots represent replicates (n = 3). Different letters indicate significant differences at *p* < 0.05 (Tukey’s test). Observations were recorded on 45 DAG.

Under drought stress, net photosynthetic rate on 14 DOT decreased by 39% in D compared with WW, whereas G+B+D, G+D, and B+D increased it to 30.2, 26.5, and 23.5 µmol CO_2_ m^-2^ s^-1^ over D, respectively (**Figure 2A**). Stomatal conductance also improved in G+B+D, G+D, and B+D, by 4.2, 3.9, and 3.5 mmol m^-2^ s^-1^, compared with 2.6 mmol m^-2^ s^-1^ in D (**Figure 2B**). Similarly, transpiration rate increased to 8.8, 7.05, and 5.7 mmol H_2_O m^-2^ s^-1^ in G+B+D, G+D, and B+D, respectively, relative to D (**Figure 2C**). Quantum yield of PSII (Fv/Fm) declined in D but was higher in the combined treatments, with values of 0.85 in G+B+D, 0.77 in G+D, and 0.59 in B+D compared with 0.51 in D (**Figure 2D**).

**Figure 3:**
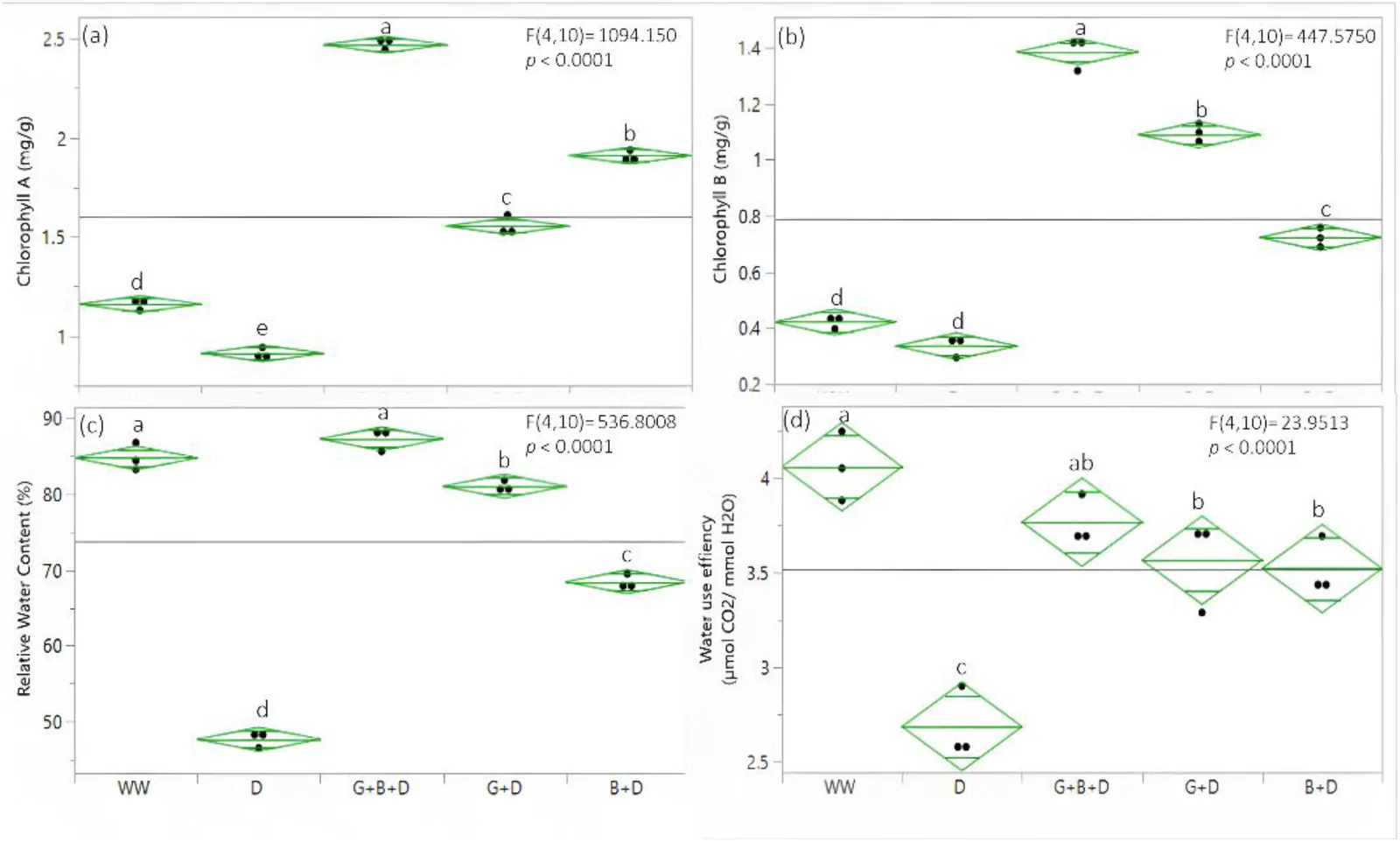
Violin plots showing the effects of grafting and biodynamic nanosilica on **(A)** chlorophyll *a*, **(B)** chlorophyll *b*, **(C)** relative water content, and **(D)** water-use efficiency in chilli plants under drought stress. Treatments include well-watered (WW), drought (D), grafted + BNS + drought (G+B+D), grafted + drought (G+D), and BNS + drought (B+D). Black dots represent replicates (n = 3). Different letters indicate significant differences at *p* < 0.05 (Tukey’s test). Observations were recorded on 45 DAG.

Relative water content (RWC) was also significantly higher in G+B+D (83%), G+D (70%), and B+D (43%) compared to D plants (**Figure 3C**). Similarly, water-use efficiency (WUE) improved by 40%, 32%, and 31% in G+B+D, G+D, and B+D treatments, respectively (**Figure 3D**). In addition, Chlorophyll *a* content increased by 2.7-, 2.1-, and 1.7-fold, while chlorophyll *b* increased by 4.1-, 2.2-, and 3.3-fold in G+B+D, G+D, and B+D treatments, respectively, compared to D plants (**Figures 3A, B**).

### 3.4 Osmolyte accumulation, phenolic enrichment, and oxidative damage under drought

**Figure 4:**
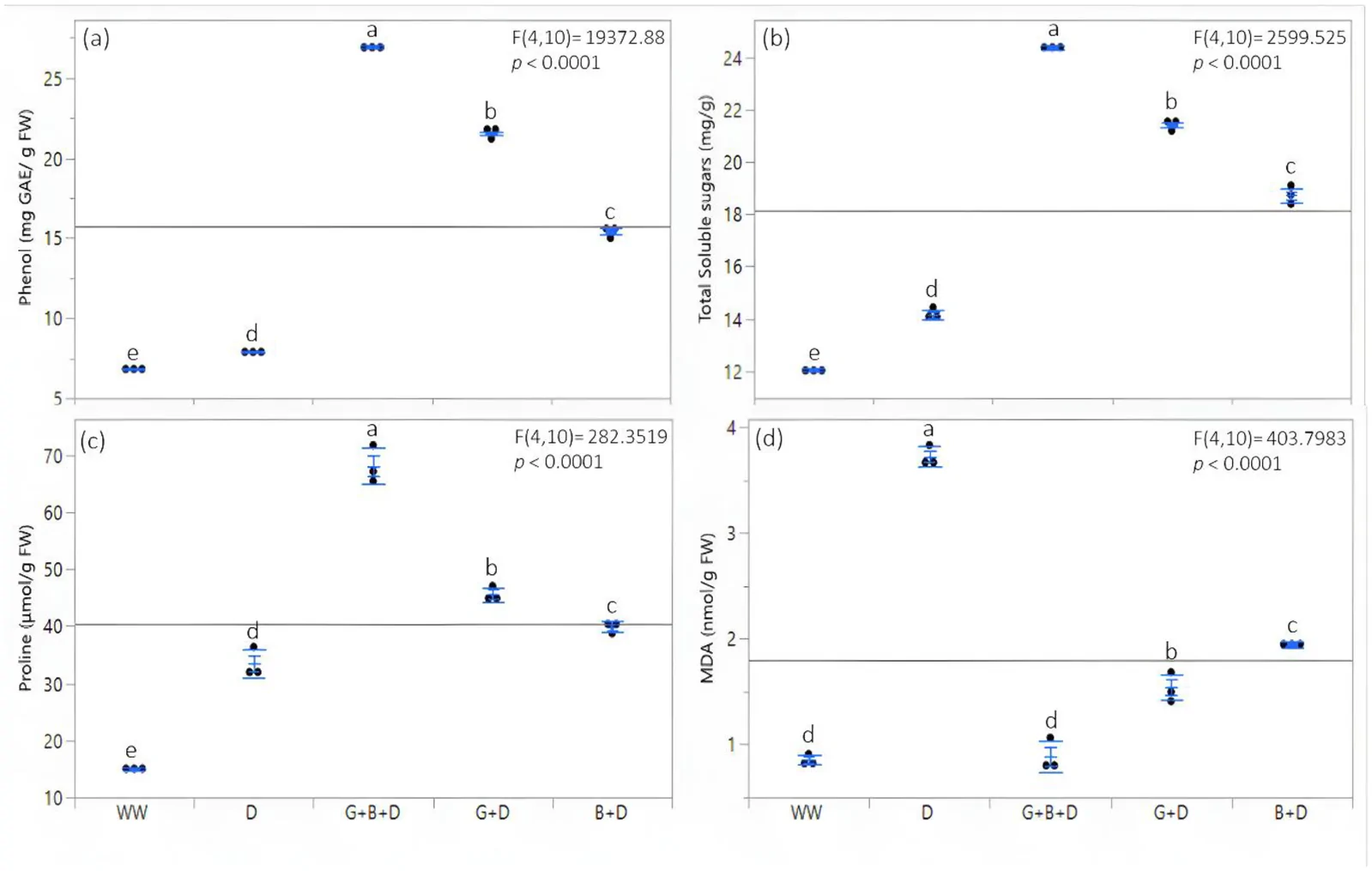
Effects of grafting and biodynamic nanosilica on **(A)** phenolics, **(B)** total soluble sugars, **(C)** proline, and **(D)** malondialdehyde (MDA) of chilli plants under drought stress. Dot plots show individual replicates (n = 3) and mean ± SE. well-watered (WW), drought (D), grafted + nanosilica + drought (G+B+D), grafted + drought (G+D), and nanosilica + drought (B+D). Different letters indicate significant differences at *p* < 0.05 (Tukey’s test).

Drought stress increased proline accumulation in chilli plants, with the highest proline content in G+B+D (68.2 μmol g^-1^ FW), followed by G+D (36%) and B+D (19%) (**Figure 4C**). Phenolic content was also significantly enhanced by all treatments under drought. Compared with D (7.8 mg GAE g^-1^ FW), phenol concentration increased to 26.9, 21.5, and 15.4 mg GAE g^-1^ FW in G+B+D, G+D, and B+D, respectively, corresponding to 3.4-, 2.7-, and 1.9-fold increases (**Figure 4A**). Soluble sugar content was increased in G+B+D (24.38 mg g^-1^), followed by G+D (21.4 mg g^-1^), with G+B+D showing a 72% increase over D (**Figure 4B**). In contrast, MDA accumulation was reduced in G+D and B+D lowering by 58% (1.5 nmol g^-1^ FW) and 47% (1.9 nmol g^-1^ FW), respectively, relative to D. The combined G+B+D treatment showed the greatest reduction in MDA, decreasing it by approximately 76% to 0.8 nmol g^-1^ FW under drought (**Figure 4D**).

Silica deposition differed significantly among treatments (**Supplementary Figure 4**). The highest silica accumulation was recorded in G+B+D and B+D (0.8 mg g^-1^ FW), whereas WW, D, and G+D showed lower values (0.2–0.3 mg g^-1^ FW). These results indicate effective uptake and tissue accumulation of BNS, with grafting further supporting silica deposition under drought stress.

### 3.5 Enzymatic antioxidant defence under drought stress

**Figure 5:**
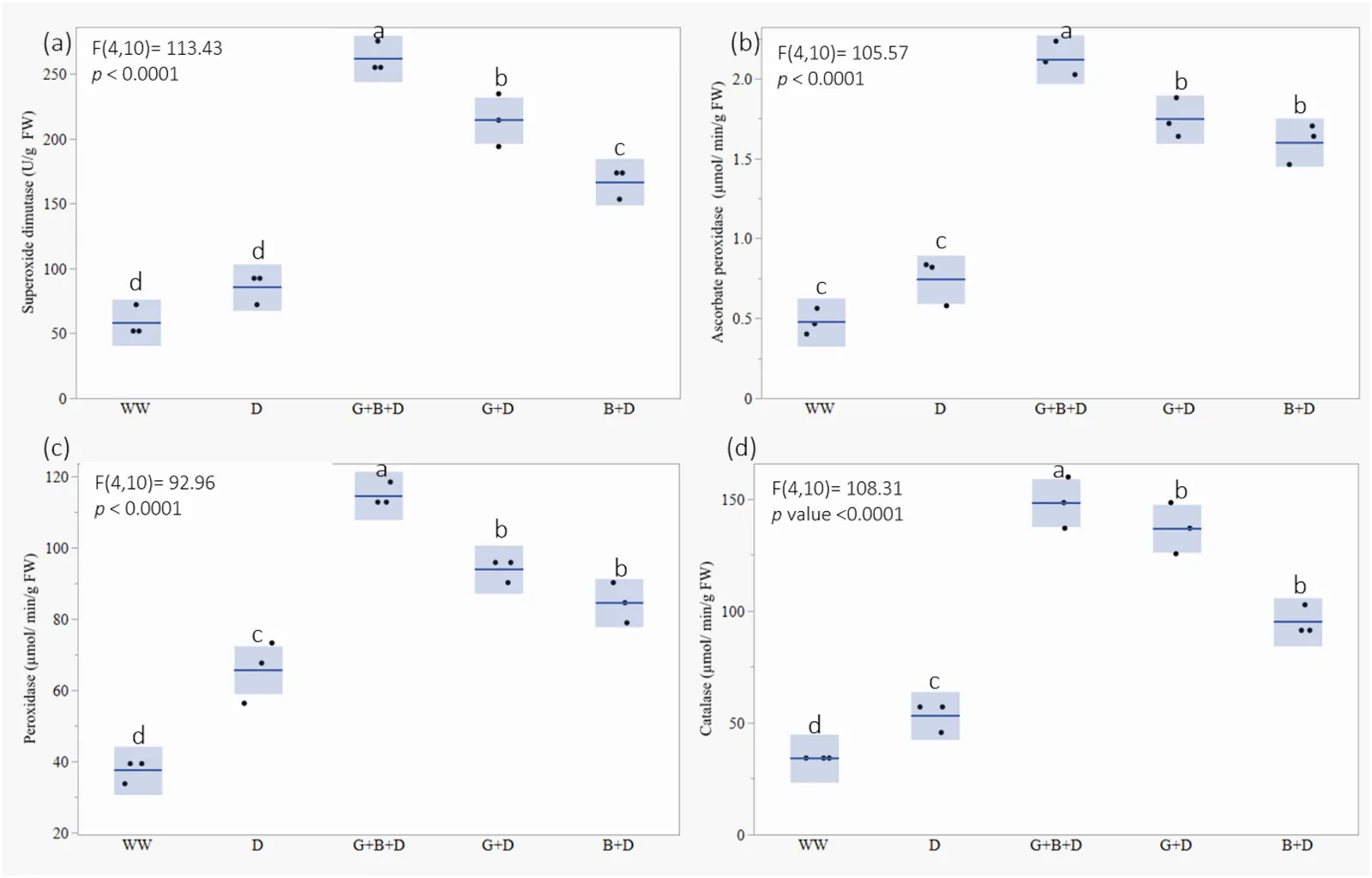
Effects of grafting and biodynamic nanosilica on **(A)** superoxide dismutase (SOD), **(B)** ascorbate peroxidase (APX), **(C)** peroxidase (POD) and **(D)** catalase (CAT) activities in chilli plants under drought stress. Box-and-whisker plots show median and interquartile range with individual replicates (n = 3) overlaid. Different letters indicate significant differences at *p* < 0.05 (Tukey’s test).

Drought stress increased antioxidant enzyme activities across all treatments, with the combined G+B+D treatment showing the strongest response. SOD activity increased from 85.6 in D to 261.6 U g^-1^ FW in G+B+D, followed by G+D (214.2 U g^-1^ FW) and B+D (166.8 U g^-1^ FW) (**Figure 5A**). POD increased from 65.8 µmol min^-1^ g^-1^ FW in D to 114.7, 94.0, and 84.6 µmol min^-1^ g^-1^ FW in G+B+D, G+D, and B+D, respectively (**Figure 5C**). CAT activity also increased by 148.5, 137.1, and 95.2 µmol min^-1^ g^-1^ FW in G+B+D, G+D, and B+D, compared with 53.3 in D (**Figure 5D**). APX followed a similar trend, increasing from 0.74 µmol min^-1^ g^-1^ FW in D to 2.12, 1.75, and 1.60 µmol min^-1^ g^-1^ FW in G+B+D, G+D, and B+D, respectively (**Figure 5B**).

### 3.6 Fruit yield and morphometric parameters under drought stress

Because fruit is the principal commercial product of chilli, yield traits were considered critical outcome variables. Drought stress drastically reduced yield parameters. Under well-water (WW) conditions, a single plant produced 41 fruits weighing approximately 144 g (**Supplementary Figure 6**). Fruit weight per plant increased from 50.18 g in D to 226.94 g in G+B+D. Similarly, fruit number per plant increased from 24.67 in D to 44.33 in G+B+D. (**Table 3**). Similarly, the fruit weight increased from 2.10 g in D to 5.14 g in G+B+D. Fruit diameter increased from 0.96 cm in D to 1.40 cm in G+B+D, 1.22 cm in G+D, and 0.93 cm in B+D. Similarly, fruit volume increased from 2.1 cm^3^ in D to 13.7 cm^3^ in G+B+D, 9.5 cm^3^ in G+D, and 3.6 cm^3^ in B+D. However, the fruit shape index was higher in WW with 7.1 cm followed by in D with 6.8 cm, and in G+D (6.7 cm), G+B+D (6.4 cm), and B+D (5.8 cm). Therefore, under drought stress, the combined grafting and BNS treatment produced the highest fruit number, fruit weight, fruit length, fruit diameter, fruit volume, and fruit dry weight relative to drought alone.

**Table 3:** Effects of grafting and biodynamic nanosilica on yield parameters in chilli plants under drought stress.

Treatments	Fruit number/plant	Fruit weight/plant (g)	Fruit length (cm)	Fruit diameter (cm)	Fruit shape index (cm)	Fresh weight/fruit (g)	Fruit volume (cm^3^)	Dry weight/fruit (g)
WW	41.00 ± 1.00b	144.25 ± 2.08c	6.90 ± 0.05c	0.96 ± 0.02c	7.17 ± 0.14a	3.59 ± 0.05c	5.06 ± 0.20c	0.46 ± 0.02c
D	24.67 ± 0.88e	50.18 ± 0.96f	5.01 ± 0.06e	0.73 ± 0.03e	6.98 ± 0.28b	2.10 ± 0.06e	2.10 ± 0.15e	0.22 ± 0.01e
G+B+D	44.33 ± 1.45a	226.94 ± 9.48a	8.93 ± 0.12a	1.40 ± 0.02a	6.40 ± 0.14d	5.14 ± 0.10a	13.70 ± 0.27a	0.72 ± 0.05a
G+D	36.67 ± 1.76c	180.71 ± 6.83d	8.17 ± 0.08b	1.22 ± 0.02b	6.72 ± 0.15c	4.93 ± 0.27b	9.56 ± 0.34b	0.62 ± 0.04b
B+D	29.67 ± 1.20d	69.34 ± 1.76e	5.42 ± 0.09d	0.93 ± 0.02d	5.86 ± 0.12e	2.38 ± 0.11d	3.67 ± 0.16d	0.31 ± 0.02d

WW, well-watered; D, drought; G+B+D, grafted + BNS + drought; G+D, grafted + drought; B+D, BNS + drought. Mean ± standard error followed by same letter/symbol(s) are not significantly different according Tukey test (*p* < 0.05).

### 3.7 Factorial analysis of grafting and nanosilica effects on chilli performance

Two-way ANOVA revealed that both grafting and biodynamic nanosilica (BNS) exerted significant effects on most morphological, physiological, biochemical, and fruit-related parameters (**Table 4**). In shoot and root morphology, grafting had a highly significant effect on plant height, leaf area, root length, surface area, root volume, root branching intensity, and root complexity. BNS significantly influenced plant height and leaf area, but its effect on most root traits was weaker or non-significant. The interaction between grafting and BNS was significant only for leaf area, whereas it remained non-significant for most root architectural traits, indicating that grafting was the primary driver of root system modification. For physiological traits, both grafting and BNS significantly improved net photosynthetic rate, stomatal conductance, transpiration rate, quantum yield, relative water content, water-use efficiency, and chlorophyll content. The interaction effect was particularly strong for net photosynthetic rate, stomatal conductance, relative water content, and water-use efficiency, showing that the combined treatment produced a response greater than that expected from either factor alone for these variables.

**Table 4:** Two-way ANOVA showing main and interaction effects of grafting and biodynamic nanosilica (BNS) on morphological, physiological, biochemical, and fruit traits in chilli under drought.

Parameters	Grafting	BNS	Grafting × BNS
Shoot and root morphology			
Plant Height	39.19***	344.09***	1.94ns
Leaf Area	217.54***	6.20*	11.25**
Root Length	76.70***	3.32	0.06
Surface Area	58.52***	0.94ns	0.14ns
Root density	1.32ns	4.05ns	3.57ns
Root volume	27.66***	0.01ns	0.78ns
Tips	0.43ns	21.49***	3.70ns
Root branching Intensity	82.93***	0.94ns	0.002ns
Root complexity	60.88***	0.43ns	0.04ns
Plant physiology			
Net Photosynthetic rate	1874.60***	840.81***	132.76***
Stomatal conductance	236.87***	49.28***	8.17*
Transpiration rate	237.37***	82.48***	0.03ns
Quantum Yield	7032.68***	797.44***	0.18ns
RWC	1373.39***	367.32***	105.89***
WUE	28.86***	24.54***	9.19*
Chlorophyll a	1039.39***	2633.90***	4.87ns
Chlorophyll b	1134.60***	263.97***	4.83ns
Biochemical parameters			
Phenol	40890.29***	10758.24***	291.93***
Sugar	4211.93***	1415.99***	64.33***
Proline	310.56***	158.76***	48.96***
MDA	770.18***	432.08***	92.53***
Silica	0.15ns	357.58***	1.97ns
SOD	194.46***	64.46***	4.46ns
POD	92.40***	42.20***	0.09ns
CAT	202.50***	30.63**	10.00**
APX	125.71***	82.45***	12.62**
Fruit parameters			
Fruit number/plant	105.26***	23.75**	1.05ns
Fruit weight/plant	716.06***	36.87***	6.32*
Fruit length	282.21***	15.02**	0.11ns
Fruit diameter	215.78***	47.67***	1.02ns
Fruit shape index	0.19ns	6.91*	2.21ns
Fruit weight/fruit	118.84***	1.56ns	0.36ns
Fruit Volume	398.80***	64.23***	5.32*
Dry Fruit weight/fruit	490.11***	28.23***	1.60ns

Values represent F-statistics from two-way ANOVA. G, grafting; BNS, biodynamic nanosilica. G × BNS indicates interaction effects. Significance levels are indicated as *p* < 0.05 (*), *p* < 0.01 (**), and *p* < 0.001 (***); ns, non-significant; RWC, relative water content; WUE, water-use efficiency; SOD, superoxide dismutase; POD, peroxidase; CAT, catalase; APX, ascorbate peroxidase.

Biochemical parameters were strongly affected by both factors. Grafting and BNS significantly influenced phenol, sugar, proline, MDA, and antioxidant enzyme activities, with BNS having especially strong effects on phenolic compounds, sugars, chlorophyll, and silica accumulation. The interaction term was significant for phenol, proline, MDA, CAT, and APX, but not for silica, SOD, or POD, indicating that the combined treatment especially altered osmoprotectant accumulation and membrane-protection-related responses. For fruit traits, grafting significantly affected all yield-related parameters. BNS also significantly influenced fruit number per plant, fruit weight per plant, fruit length, fruit diameter, fruit volume, and dry fruit weight per fruit, although it had no significant effect on fruit shape index and fruit weight per fruit. The grafting × BNS interaction was significant for fruit weight per plant, fruit volume, and dry fruit weight per fruit, but non-significant for most other fruit traits, suggesting that the combined treatment particularly enhanced overall reproductive output and biomass accumulation.

### 3.8 Correlation analysis of physiological and antioxidant traits under drought

Correlation analysis revealed strong functional linkages between physiological, biochemical, and antioxidant traits (**Supplementary Figure 7**). Chlorophyll a was positively correlated with relative water content (r = 0.57), net photosynthetic rate (r = 0.80), superoxide dismutase (r = 0.86), and catalase (r = 0.80) and negatively correlated with malondialdehyde (r = 0.56), indicating reduced oxidative damage with improved photosynthetic capacity. RWC was strongly positively correlated with net photosynthesis (r = 0.93) and strongly negatively correlated with MDA (r = -0.98), suggesting a central role of plant water status in mitigating lipid peroxidation. Proline accumulation was positively associated with SOD (r = 0.93) and CAT (r = 0.91), supporting its role in stress adaptation in the cells. MDA exhibited strong negative correlations with net photosynthesis (r = -0.91) and antioxidant activity, are consistent with the harmful effects of oxidative stress on photosynthetic performance. Net photosynthesis was positively correlated with both SOD (r = 0.72) and CAT (r = 0.68), and a very strong positive correlation (r = 0.98) between SOD and CAT suggested coordinated antioxidant regulation.

### 3.9 Transcriptome profiling and functional enrichment of drought-silica-responsive genes

The volcano plot shows the global distribution of differentially expressed genes (DEGs) between the G+B+D and D treatments under drought stress (**Supplementary Figure 8**). A total of 1051 DEGs were identified (FDR < 0.05, |log_2_FC| ≥ 1), comprising 431 upregulated and 620 downregulated genes. Approximately 250 genes showed strong differential expression (|log_2_FC| > 2, adjusted p < 0.01), indicating a strong transcriptional divergence between the two treatments.

The Venn diagram shows the distribution of expressed genes between the G+B+D and D treatments under drought stress (**Supplementary Figure 9**). G+B+D identified 10551 genes and D identified 10493 genes. Among them, 431 genes were uniquely expressed only in G+B+D and 373 genes in D. Moreover, 10120 was commonly present in both the G+B+D and D treatments.

**Figure 6:**
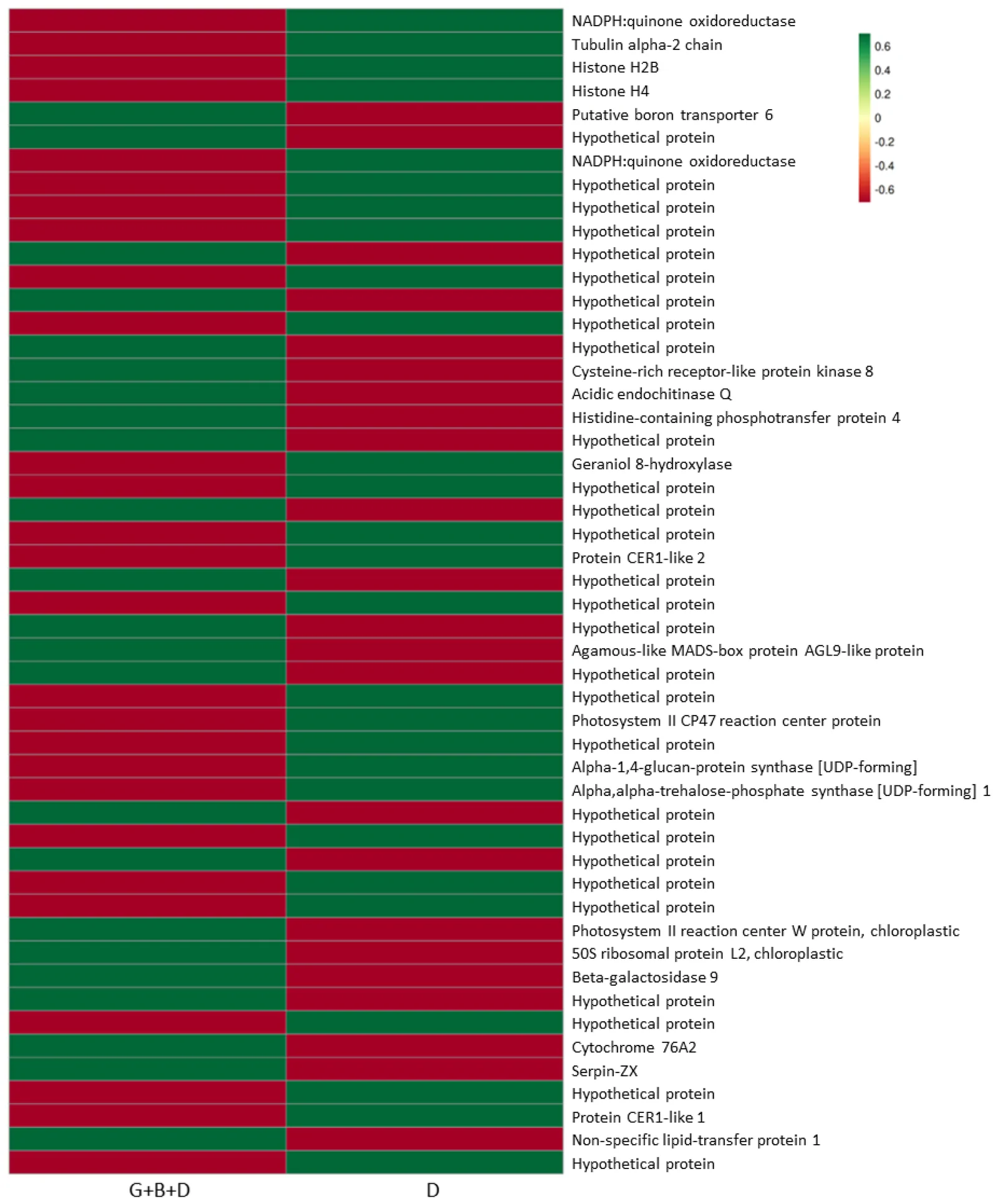
Heatmap showing hierarchical clustering of the top 50 differentially expressed genes between G+B+D and D treatments under drought stress.

Hierarchical clustering of the top 50 DEGs clearly separated the G+B+D and D samples, demonstrating distinct transcriptional profiles under drought stress (**Figure 6**). The upregulated genes in G+B+D were associated with stress signalling, redox-related regulation, photosynthetic processes, and secondary metabolism. In contrast, D showed higher expression of cytoskeletal proteins (tubulin chains), histone-associated proteins, and selected cytochrome P450 family members, which were relatively downregulated in G+B+D. The contrasting expression patterns revealed treatment-dependent differences in drought-responsive gene expression.

GO enrichment analysis revealed a significant abundance of biological processes in response to stimuli, stress, and defence responses (**Supplementary Figure 10**). In addition, the GO terms associated with cell cycle regulation, mitotic processes, chromosome segregation, and DNA replication were enriched. The enrichment of stress-related and cell cycle–associated processes showed coordinated transcriptional regulation under drought stress in G+B+D-treated plants.

**Figure 7:**
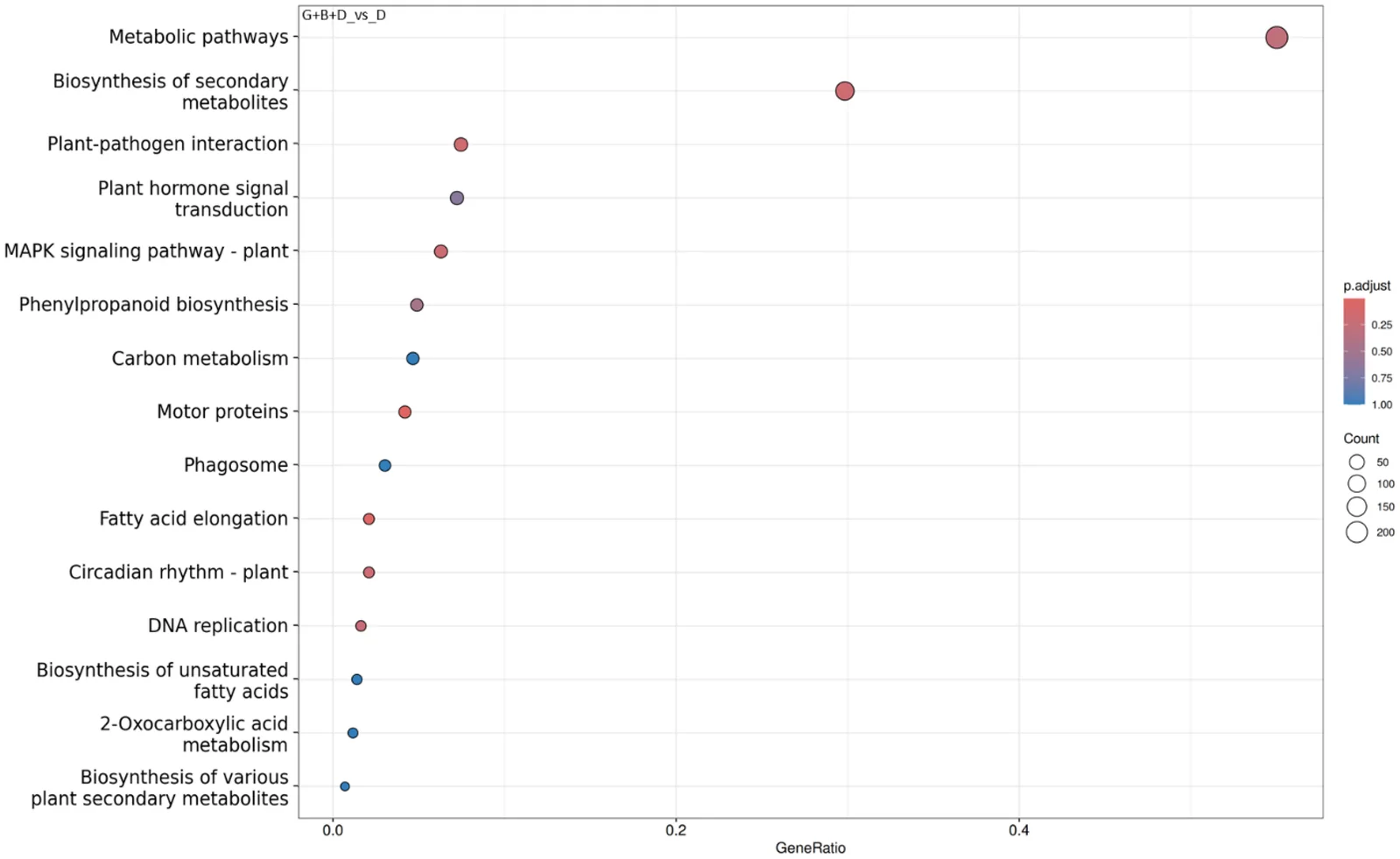
KEGG pathway enrichment analysis of DEGs between G+B+D and D treatments under drought stress.

KEGG pathway analysis identified significant enrichment of plant–pathogen interaction, MAPK signalling, and phenylpropanoid biosynthesis pathways (**Figure 7**). Additionally, enrichment was observed in carbon metabolism, secondary metabolite biosynthesis, and proteasome-related pathways. These enriched pathways suggest potential involvement of stress signalling modulation, defence response and metabolic processes in G+B+D under drought stress. However, functional validation will be required to confirm these pathway-level inferences.

Molecular function enrichment analysis revealed strong representation of abscisic acid–related activities, including abscisic acid binding and (+)-abscisic acid 8-hydroxylase activity. Protein phosphatase regulator and activator activities, along with protein kinase activity, were prominently enriched, indicating extensive modulation of phosphorylation-based signalling (**Supplementary Figure 11**). Additionally, enrichment of superoxide dismutase activity and heme transmembrane transporter activity suggests enhanced redox regulation and stress-associated transport processes under drought conditions.

### 3.10 Protein–protein interaction network of drought- silica -responsive genes in grafted and BNS-treated plants

**Figure 8:**
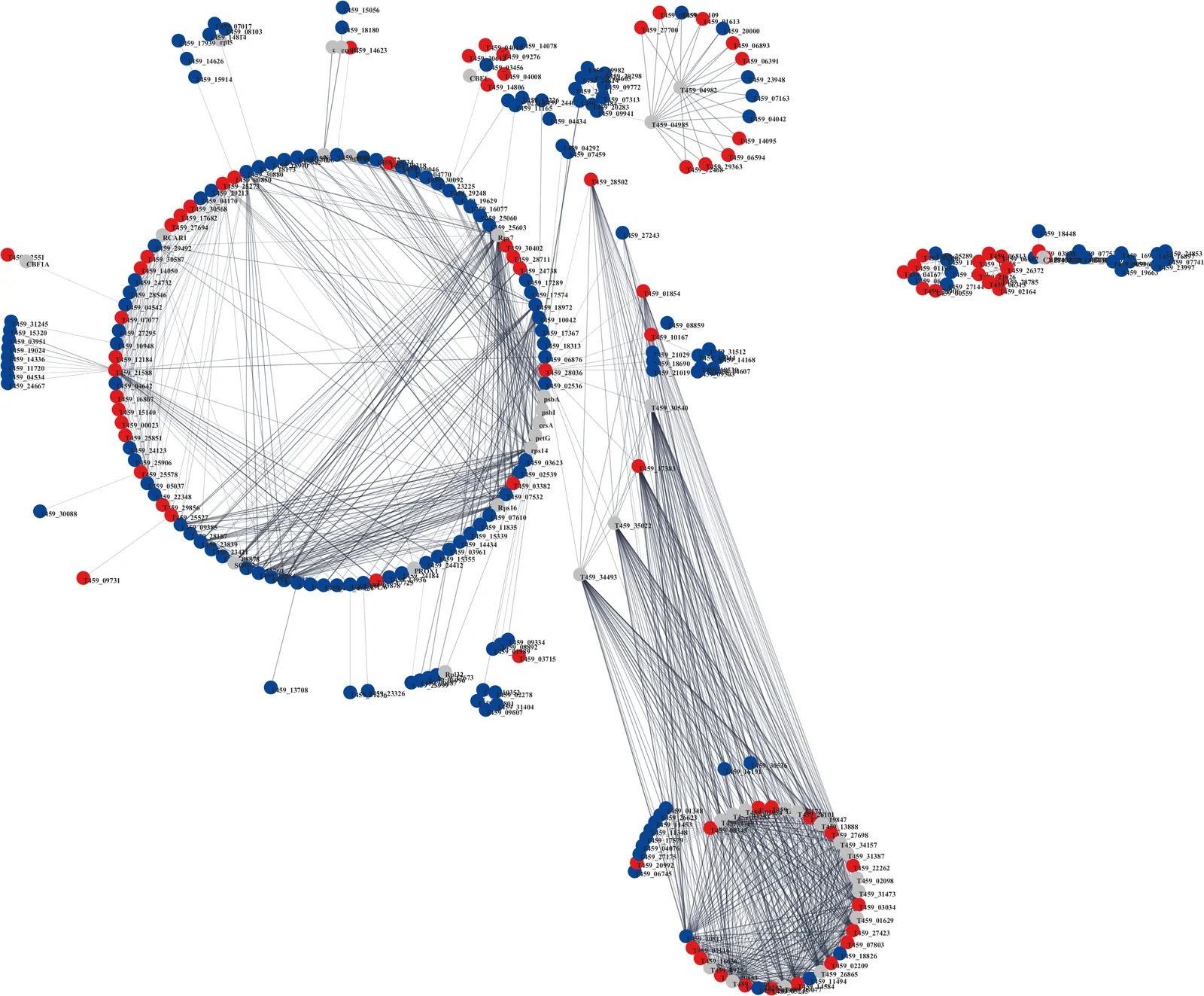
Protein–protein interaction (PPI) network of drought-, grafting-, and nanosilica-responsive genes. Nodes represent proteins, and edges indicate known or predicted protein–protein interactions. Node colours denote treatment specificity: drought + nanosilica-responsive genes (red), grafting-associated genes (blue), and genes shared among drought, nanosilica, and grafting responses (grey).

To gain a better understanding of the biological functions and interactions of the genes identified in our study (gene ID with gene description attached as **Supplementary Table 1**), we constructed a protein-protein interaction network using the STRING database (**Figure 8**). This interaction analysis suggested a highly interconnected graft and drought-responsive network dominated by a central core network. The dense connectivity within major clusters suggests coordinated regulation of stress-responsive signalling pathways. Grafting-associated genes were embedded within this core and in secondary clusters rather than forming isolated modules. Several shared genes connected drought- and grafting-responsive clusters, may associated with functional integration of grafting signals into the drought stress regulatory network. However, nanosilica-responsive genes including aquaporins, antioxidant enzymes, ABA signalling components, and cell wall–associated proteins, may associated within the core network, suggests that nanosilica supplementation strengthens drought adaptation by enhancing water transport, redox homeostasis, hormonal signalling, and structural resilience. This result is considered as preliminary, further validation experiments needed.

## 4 Discussion

### 4.1 Shoot growth and root architecture under drought

Drought constrains vegetative development by reducing turgor pressure, suppressing meristematic activity, and limiting carbon assimilation. These consequences were clearly reflected in the severe shoot and root suppression observed in D plants. The treatment-dependent recovery of plant height and leaf area, ranking G+B+D > G+D > B+D > D, indicates that grafting was the primary factor sustaining vegetative growth under drought, while BNS provided additional support when applied in combination. Rootstock-mediated growth maintenance is likely attributable to improved hydraulic conductance through the graft union, which may preserve the turgor required for cell elongation under declining soil water potential. BNS may have supported this response through foliar silica deposition. Silicon has been associated with epidermal cell wall strengthening and reduced cuticular water loss in horticultural crops under water deficit (Daler et al., 2025; Naik et al., 2024). The susceptibility of non-grafted chilli to sustained drought without silicon supplementation is consistent with observations in non-grafted pepper under comparable deficit conditions (Gisbert-Mullor et al., 2020; Trejo-Paniagua et al., 2024). The larger leaf area retained in treated plants is functionally relevant, as it may support continued carbon fixation and dry matter accumulation under stress (Ghahremani et al., 2023).

Root architecture provided stronger evidence of drought adaptation than shoot parameters. The greater root length, volume, branching intensity, and structural complexity in G+B+D and G+D indicate that grafting promoted more extensive soil exploration under drought. Drought-tolerant rootstocks are known to reshape root biomass distribution, improving water acquisition under deficit irrigation (Bahadur et al., 2024; Dogan et al., 2025a; Kappel et al., 2024; Zhang et al., 2020). The elevated root tip density in B+D relative to D points to a possible independent effect of BNS on fine root initiation, potentially through silicon’s role in root epidermal cell elongation and stabilisation (Bekkam and Thiyagarajan, 2024). Furthermore, root density remained uniform across all experimental units (P > 0.05) and treated root systems expanded beyond WW baseline levels, the observed architectural enhancements (**Table 2**) reflect intrinsic genetic plasticity rather than container-induced growth limitations. Although biological replication was limited (n = 3), the observed trends were consistent across multiple parameters; nevertheless, further validation with greater replication would strengthen confidence in the results.

### 4.2 Photosynthetic performance, stomatal regulation, and water relations under drought

Grafting and BNS helped sustain photosynthetic performance, stomatal regulation, and leaf hydration under drought, as reflected in higher net photosynthetic rate, stomatal conductance, transpiration, PSII efficiency, relative water content, and water-use efficiency in G+B+D, G+D, and B+D relative to D (Dilnawaz et al., 2023). Rootstock-enhanced hydraulic conductance likely contributed to stomatal aperture maintenance by preserving leaf turgor, a linkage well documented in grafted pepper under water stress (López-Serrano et al., 2019; Padilla et al., 2021; Gara-Padilla et al., 2023). Silica has also been associated with stomatal regulation and cellular water retention, which may have contributed to the elevated WUE observed in BNS-treated plants (Bolat et al., 2024; Daler et al., 2025; Dogan et al., 2025b; Ulas et al., 2025).

The reduced Fv/Fm in D plants are consistent with drought-induced photoinhibition and oxidative damage to PSII reaction centres. Its relative protection in treated plants indicates reduced oxidative pressure at the thylakoid level, a response that aligns with the antioxidant enzyme induction discussed in Section 4.3. Chlorophyll a and b were also better maintained in treated plants, indicating lower pigment degradation and improved thylakoid membrane stability. Silicon-mediated oxidative protection has been associated with similar outcomes in drought-stressed crops (Daler et al., 2025; Pandey et al., 2026). The stronger physiological performance of G+B+D across all parameters points to a complementary action between grafting and BNS, though the precise interaction warrants further investigation under field conditions.

### 4.3 Osmotic adjustment, antioxidant defence, and oxidative stress mitigation under drought

Proline, soluble sugars, and phenolic compounds accumulated progressively in treated plants, reflecting coordinated osmotic adjustment and antioxidant activation under drought. Proline was highest in G+B+D. It functions as a compatible solute and ROS scavenger, and its greater accumulation in BNS-treated plants may reflect silicon-associated activation of proline biosynthetic pathways reported in nano-silicon studies under drought (Mahabadi et al., 2026; Padilla et al., 2024; Seyed Hajizadeh et al., 2023). Soluble sugars provided complementary osmotic protection, likely contributing to membrane stability under declining water activity (Etesami et al., 2022; Sayed et al., 2022). Phenolic enhancement in grafted and BNS-treated plants indicates activation of secondary metabolism. Phenolics contribute to direct ROS scavenging and serve as substrates amplifying peroxidase-catalysed H_2_O_2_ detoxification, a linkage elaborated further below (Desoky et al., 2025; Iqbal et al., 2023).

MDA content was lower in all treated plants than in D, indicating reduced lipid peroxidation and improved membrane protection. ROS were not directly quantified in this study, so oxidative stress mitigation is inferred indirectly from MDA trends and enzyme activities. This interpretive approach is consistent with nano-silica drought studies reporting similar evidence of redox improvement (Daler et al., 2024, 2025; Esmaili et al., 2022). Silica accumulation confirmed in G+B+D and B+D leaf tissues demonstrates effective BNS uptake, may provide structural support for the observed protective effects through cell wall reinforcement and epidermal silicic acid deposition (Daler et al., 2025; Bekkam and Thiyagarajan, 2024; Mushtaq et al., 2024).

SOD activity was highest in G+B+D and declined progressively toward D. Its co-elevation with proline, phenolics, and soluble sugars with reduced MDA, points to a multi-component antioxidant response rather than isolated enzyme induction (Das et al., 2023; Padilla et al., 2025; Yang et al., 2025). The elevated POD activity in G+B+D likely reflects the availability of phenolic substrates for H_2_O_2_ detoxification, connecting non-enzymatic and enzymatic defence (Rao and Zheng, 2025). CAT activity was inversely associated with MDA across treatments, indicating improved H_2_O_2_ decomposition that may have limited membrane peroxidation (Kumar, 2024; Rao et al., 2024). APX induction in G+B+D is consistent with chloroplastic H_2_O_2_ removal through the ascorbate-dependent pathway, aligning with the Fv/Fm and chlorophyll trends reported in Section 4.2, though a direct causal relationship requires targeted validation (Noctor, 2025). The concurrent induction of all four enzymes in G+B+D, alongside non-enzymatic defences, indicates an antioxidant response in the combined treatment than in either intervention applied alone (Bolat et al., 2024; Nazir et al., 2022; Shehata et al., 2022).

### 4.4 Yield performance under drought

Drought severely reduced fruit number, weight, length, diameter, volume, and dry weight in D plants, reflecting impaired hydraulic supply, reduced photosynthetic carbon fixation, and elevated oxidative stress during reproductive development. Grafting and BNS mitigated these losses in a treatment-dependent manner, with G+B+D consistently recording the highest values across all yield components. Rootstock-mediated water supply, maintained photosynthetic activity, and reduced oxidative load are plausible contributors to better fruit set and expansion in grafted treatments, in accordance with reports of grafting-mediated yield protection under drought in pepper (Daler et al., 2025; Gisbert-Mullor et al., 2023; Murcia Asensi et al., 2024).

The improved fruit morphometric parameters in treated plants indicate that water deficit was moderated sufficiently to permit cell expansion during fruit development, which depends on adequate turgor and assimilate import into pericarp tissue (Gisbert-Mullor et al., 2020; Sae-Tang and Nawata, 2019). The lower reproductive performance of B+D relative to G+D indicates that BNS alone provides limited drought mitigation for yield, which is contrast to previous study Boora et al. (2023). Therefore rootstock-mediated belowground adaptation remains the more influential factor for sustaining fruit production under deficit. The superior outcome in G+B+D points to a complementary interaction between grafting and BNS, though the mechanistic basis has not been directly established here. Field-scale validation is required before these findings can inform broader production recommendations.

### 4.5 Transcriptomic regulation under drought

Transcriptome profiling of G+B+D relative to D identified 431 upregulated and 620 downregulated DEGs. Pathway enrichment analysis indicated over-representation of MAPK signalling, ABA metabolic and binding functions, phenylpropanoid biosynthesis, and redox regulatory processes. These enriched pathways are broadly consistent with the physiological and biochemical observations reported in this study: MAPK signalling corresponds to stress signal transduction; ABA pathway enrichment is concordant with the stomatal and osmotic responses observed; phenylpropanoid enrichment is consistent with the elevated phenolic content measured biochemically; and redox pathway representation aligns with the antioxidant enzyme activities reported in Section 4.3 (Muhammad Aslam et al., 2022; Lim et al., 2020; Zhu et al., 2020).

To further interpret the biological significance of the transcriptomic findings, an integrated transcriptomic–phenotypic concordance analysis was performed, in which the directional patterns of DEG expression were interpreted alongside independently measured biochemical and physiological responses obtained from the same plant tissue at the same sampling time (45 DAG). This approach evaluates whether transcriptional patterns are consistent with observed phenotypic outcomes, but does not establish mechanistic relationships between individual genes and measured traits.

The combined transcriptomic, biochemical, and physiological datasets showed concordant responses across multiple drought-responsive pathways. Multiple transcriptomic–phenotypic concordances were observed between DEG expression patterns and measured outcomes (**Supplementary Table 2**). Upregulation of phenylpropanoid biosynthesis-related genes, including Chalcone synthase 2 (log_2_FC = +8.03, FDR < 0.0001) and Phenylalanine N-monooxygenase (log_2_FC = +8.90, FDR < 0.0001), was observed alongside the measured 3.4-fold increase in total phenolic content in G+B+D (26.9 vs. 7.8 mg GAE g^-1^ FW in D). Upregulation of redox-associated genes including Monothiol glutaredoxin-S2 (log_2_FC = +8.32) and Peroxidase 5 (log_2_FC = +5.72) was coincided with higher SOD (3.1-fold), CAT (2.8-fold), POD, and APX activities and a 76% reduction in MDA in G+B+D relative to D. Upregulation of ABA-responsive genes including Abscisic stress-ripening protein 3 (log_2_FC = +3.87) and the SnRK2-type kinase SAPK6 (log_2_FC = +8.38) was observed concurrently with improved stomatal conductance and higher relative water content.

Upregulation of aquaporin genes, specifically Aquaporin TIP1-2 (log_2_FC = +6.87) and Aquaporin NIP1-1 (log_2_FC = +3.87), was consistent with higher leaf water status in G+B+D. Osmoprotectant pathway gene expression, including upregulation of Trehalose-phosphate phosphatase B (log_2_FC = +3.46) and a stress-responsive transcription factor NF-YA8 (log_2_FC = +3.47), was concordant with the highest measured proline content (68.2 µmol g^-1^ FW) and soluble sugar accumulation (+72% over D). Finally, upregulation of MAPK cascade kinase genes including Calcium-dependent protein kinase 2 (log_2_FC = +9.73) and SAPK6 (log_2_FC = +8.38) was observed together with improvements in physiological stress responses and the higher fruit yield recorded in G+B+D plants. These observations indicate that the enriched transcriptomic pathways were accompanied by corresponding physiological and biochemical responses, providing complementary phenotypic support for the biological relevance of the RNA-seq findings while remaining distinct from direct gene-expression validation.

**Figure 9:**
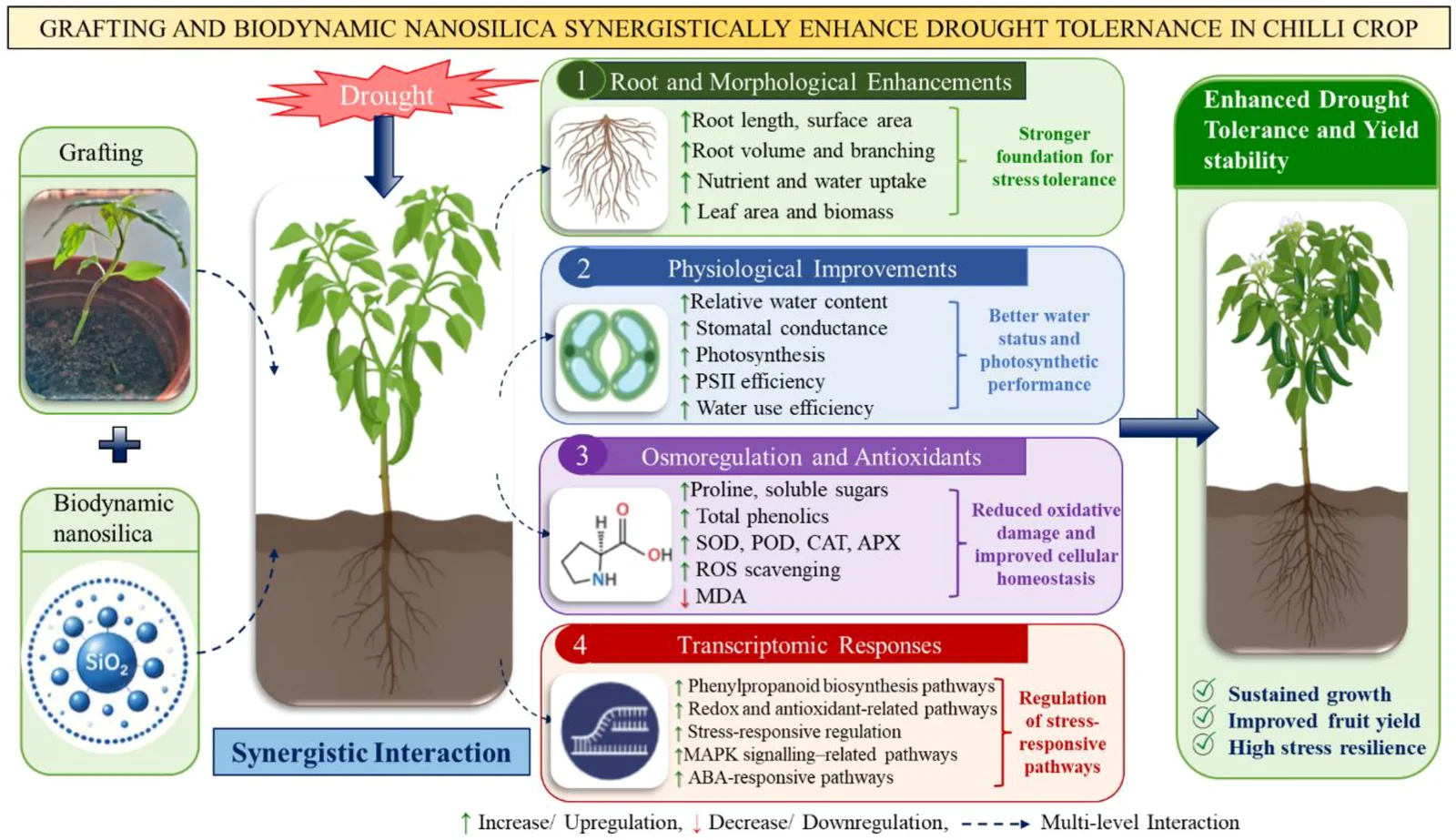
Schematic model illustrating the synergistic effects of grafting and biodynamic nanosilica (BNS) on drought tolerance in chilli through morphological, physiological, biochemical, and transcriptomic responses.

PPI network analysis indicated that grafting- and BNS-responsive DEGs tend to cluster within shared stress-associated functional modules, suggesting some degree of regulatory convergence rather than entirely independent transcriptional responses. Nanosilica-responsive gene modules related to water transport, cell wall modification, and ABA signalling appeared within these networks, which may partially account for the physiological overlap between BNS and grafting effects (Ahammed et al., 2020; Wang et al., 2021). Based on the integrated physiological, biochemical, and transcriptomic responses observed in this study, a schematic model summarising the proposed drought tolerance pathways in grafted BNS-treated plants is presented in **Figure 9**.

## 5 Conclusion

This study demonstrates that grafting combined with biodynamic nanosilica is an effective strategy for improving drought tolerance in chilli. Compared with D plants, the combined treatment G+B+D enhanced shoot growth, root system architecture, photosynthetic performance, leaf water status, and water-use efficiency, thereby supporting plant productivity under drought. The treatment also promoted osmotic adjustment through increased proline, soluble sugars, and phenolic compounds, while reducing lipid peroxidation and maintaining membrane stability. Similarly, higher silica deposition and antioxidant enzyme activities indicate that grafting and biodynamic nanosilica strengthened structural protection and redox homeostasis, improving the plant’s capacity to withstand drought-induced oxidative stress.

These physiological and biochemical improvements were reflected in yield, as G+B+D significantly increased fruit number, fruit size, fruit biomass, and total yield under drought. Integrated transcriptomic–phenotypic concordance analysis revealed enrichment of MAPK signalling, ABA-mediated regulation (including ABA binding and (+)-ABA 8’-hydroxylase activity), and phenylpropanoid biosynthesis as the enriched pathways. These findings indicate that integrating grafting with biodynamic nanosilica offers a climate-smart and promising approach for stabilizing chilli production under drought conditions. Future studies should validate candidate genes, test performance under field conditions, optimize nanosilica dose across genotypes, and examine long-term effects on fruit quality and production stability.

## Data Availability

The original contributions presented in the study are publicly available. This data can be found in the NCBI repository: https://ncbi.nlm.nih.gov/bioproject/PRJNA1510951.
