# Supplementary material for: Grafting and biodynamic nanosilica-induced physiological and transcriptomic modulation of chilli (Capsicum annuum L.) under drought stress
Source: Front Plant Sci. 2026 Aug 25;17:1916999. doi: 10.3389/fpls.2026.1916999 (PMC13549884; doi:10.3389/fpls.2026.1916999)
Supplement: Supplementary file 1 [file Supplementaryfile1.docx]

**Supplementary files**

Supplementary Figure 1. Pictorial representation of experimental setup in the greenhouse.

Supplementary Figure 2. FTSW–NTR relationships under different treatments during progressive drought. Drought and B+D showed declining NTR with decreasing FTSW, whereas G+D and G+B+D modified the transpiration response, indicating treatment-dependent regulation of drought progression.

Supplementary Figure 3. Influence of grafting and biodynamic nanosilica (BNS) application on *Capsicum annuum* varieties grown across treatments: well-watered (WW), drought (D), grafted + nanosilica

Supplementary Figure 4. Effect of grafting and biodynamic nanosilica on silica deposition in chilli plants under drought stress. The dot plot shows individual replicates (n = 3) across treatments: well-watered (WW), drought (D), grafted + nanosilica + drought (G+B+D), grafted + drought (G+D), and nanosilica + drought (B+D). Different letters indicate significant differences at *p* < 0.05 (Tukey's test).

Supplementary Figure 5. Influence of grafting and BNS application on root architecture of *Capsicum annuum* varieties grown under across treatments: well-watered (WW), drought (D), grafted + nanosilica + drought (G+B+D), grafted + drought (G+D), and nanosilica + drought (B+D).

Supplementary Figure 6. Influence of grafting and BNS application on root architecture of *Capsicum annuum* varieties grown under across treatments: well-watered (WW), drought (D), grafted + nanosilica + drought (G+B+D), grafted + drought (G+D), and nanosilica + drought (B+D).

Supplementary Figure 7. Correlation matrix between physiological and antioxidant enzymes in chilli plants under drought stress.

Supplementary Figure 8. Volcano plot showing the distribution of differentially expressed genes between G+B+D (grafted + BNS + drought) and D (drought) chilli plants under drought stress.

Supplementary Fig 9. Venn diagram shows the distribution of expressed genes between G+B+D and D treatments under drought stress.

Supplementary Figure 10. GO enrichment analysis of DEGs identified between G+B+D and D under drought stress

Supplementary Figure 11. Molecular function enrichment of drought-responsive genes. Bubble plot showing significantly enriched Gene Ontology (GO) molecular function terms. The x-axis represents enrichment strength, bubble size indicates the number of genes per term, and colour denotes false discovery rate (FDR).

Supplementary Table 1. Summary of raw and clean read statistics for RNA-seq data of chilli under drought and combined grafting + biodynamic nanosilica (G+B+D) treatments

| Sample | total no of reads | total no of bases | Q20(%) | Q30(%) | Data in GB |
| --- | --- | --- | --- | --- | --- |
| RAW READ STATISTICS | | | | | |
| G+B+D | 51849214 | 7808595682 | 93.83 | 87.66 | 7.808595 |
| D | 39403758 | 5934643226 | 92.9 | 86.49 | 5.934643 |
| CLEAN READ STATISTICS | | | | | |
| G+B+D | 47048388 | 6300591516 | 98.18 | 93.7 | 6.300591 |
| D | 35170676 | 4442752490 | 98.16 | 93.66 | 4.442752 |

Sequencing quality metrics, including total reads, bases, Q20, Q30, and data volume (GB), for raw and clean reads. Q20 and Q30 indicate the percentage of bases with scores ≥20 and ≥30, respectively. Clean reads were obtained after quality filtering and adapter removal.

Supplementary Table 2: Summary of Transcriptomic–Phenotypic Cross-Validation in G+B+D vs. D

| **Transcriptomic Signal (DEG)** | **Key Upregulated Gene(s) (log2FC, FDR)** | **Biochemical/ Physiological Outcome Measured** | **Measured Value in G+B+D** |
| --- | --- | --- | --- |
| **Phenylpropanoid biosynthesis** | *Chalcone synthase 2 (+8.03***) Phenylalanine N-monooxygenase (+8.90***) Peroxidase 5 (+5.72***)* | Total phenolic content (Folin–Ciocalteu assay) | 26.9 mg GAE g^-1^ FW (3.4-fold over D) |
| **Antioxidant/redox regulation** | *Monothiol glutaredoxin-S2 (+8.32***) Glutaredoxin-S1 (+6.78**) Peroxidase 5 (+5.72***)* | SOD, CAT, POD, APX enzyme activities + MDA (lipid peroxidation) | SOD: 3.1-fold ↑ CAT: 2.8-fold ↑ MDA: 76% ↓ |
| **ABA signalling and stomatal regulation** | *Abscisic stress-ripening protein 3 (+3.87**) SAPK6/SnRK2 (+8.38***)* | Stomatal conductance and Relative water content | Gs: 4.2 mmol m^-2^ s^-1^ RWC: 83% WUE: +40% |
| **Aquaporin / water transport** | *Aquaporin TIP1-2 (+6.87**) Aquaporin NIP1-1 (+3.87***)* | Leaf relative water content (RWC) | RWC: 83% vs 49% in D (+34%) |
| **Osmoprotectant accumulation** | *Trehalose-phosphate phosphatase B (+3.46**) NF-YA8 transcription factor (+3.47***)* | Proline and soluble sugar content | Proline: 68.2 µmol g^-1^ Sugars: +72% over D |
| **MAPK stress signalling cascade** | *CDPK2 (+9.73***) SAPK6 (+8.38***) CAMK2 (+10.04***)* | Multi-parameter stress protection & fruit yield | Photosynthesis: 2.0-fold ↑ Yield: +79% over D |

*** FDR < 0.001; ** FDR < 0.01; * FDR < 0.05. *SAPK6 = stress-activated protein kinase 6 (SnRK2-type); CDPK2 = calcium-dependent protein kinase 2; CAMK2 = calmodulin-binding receptor-like cytoplasmic kinase 2; NF-YA8 = nuclear factor Y subunit A-8; TIP = tonoplast intrinsic protein; NIP = nodulin 26-like intrinsic protein*.
